# The association between work interval regularity and sleep regularity: a 2-week observational study in daytime employees

**DOI:** 10.1093/joccuh/uiae009

**Published:** 2024-02-14

**Authors:** Hiroki Ikeda, Tomohide Kubo

**Affiliations:** Japan Organization of Occupational Health and Safety, National Institute of Occupational Safety and Health, Nagao 6-21-1, Tama-Ku, 214-8585, Kawasaki, Japan; Japan Organization of Occupational Health and Safety, National Institute of Occupational Safety and Health, Nagao 6-21-1, Tama-Ku, 214-8585, Kawasaki, Japan

**Keywords:** work interval system, daily rest period, social rhythms

## Abstract

**Objectives:** Several health issues are associated with irregular sleep patterns. However, it is unclear what causes workers to sleep irregularly. The work interval (WI) between the end of one day’s working hours and the start of the next day’s working hours contains sleep opportunities, and an irregular WI may result in irregular sleep. This study investigated this association among Japanese daytime workers.

**Methods:** This study recruited 141 daytime workers without shiftwork for a 14-day observational study. Participants reported the WI duration, WI timing, time in bed (TIB: difference between bedtime and wake-up time), and bedtime timing every day before bedtime. The SD over 14 days was used to calculate the regularity scores. Logistic regression analysis was performed. The dependent variables were ≥60 minutes of TIB SD and bedtime timing SD, whereas the independent variables were WI duration and timing SD.

**Results:** The odds ratios (ORs) (95% CIs) for ≥60 minutes of TIB SD across categories of WI duration SD were 1.000 (reference) for <30 minutes, 1.344 (0.337-5.360) for 30-60 minutes, and 4.743 (1.441-15.607) for ≥60 minutes. The ORs (95% CIs) for ≥60 min of bedtime timing SD across categories of WI timing SD were 1.000 for <30 minutes, 4.154 (1.574-10.965) for 30-60 minutes, and 7.714 (2.124-28.015) for ≥60 minutes.

**Conclusions:** Regularity of WI was associated with regularity of sleep. To ensure worker health, workers should have regular WI, and if they are exposed to irregular WI, they should make every effort to maintain regular sleep.

## Teaser

**Key points** What is already known on this topic: Irregular sleep patterns are associated with several health issues, but it is unclear what causes workers to sleep irregularly.

**What this study adds:** Regularity of work interval duration and timing was related to regularity of sleep. This result was also found among workers who had not experienced work interval durations of <11 hours.

**How this study might affect research, practice, or policy:** Although the European Union working time directive or the work interval system in Japan can guarantee a specific work interval duration, irregular sleep may not be prevented. To protect worker health, workers should maintain regular work intervals, and if they experience irregular work intervals, they may need to maintain as regular a sleep schedule as possible.

## 1. Introduction

It is well known that insufficient quantity and quality of sleep is associated with health issues, such as cardiovascular disease (CVD)^1–3^ and mental health disorders.^4^^,^^5^ In addition, it was recently reported that sleep regularity is also a crucial factor linking to health issues. For example, irregular sleep may be associated with CVD risks,^6^ a high prevalence of hypertension and an increase in blood pressure,^7^^,^^8^ a decline in metabolic health,^9^^,^^10^ and an increase in acute suicidal ideation.^11^ As a result, in addition to the quantity and quality of sleep, sleep regularity may also be important to ensure health at work.

Working hours may be one of the most influential factors for sleep regularity for employees. For example, sleep/wake schedules tend to differ between the workday, which reflects the social clock, and the nonworkday, which reflects the biological clock. This phenomenon is referred to as social jetlag^12^ and affects workers’ health^13^ and performance.^14^ Additionally, shiftwork influences sleep regularity. The work schedules of shift workers vary daily, and those who work the night shift must work when they would normally be sleeping. This causes irregular sleep^15^ and adverse effects on workers’ health.^16^^,^^17^ However, irregular sleep also occurs in spite of the absence of shiftwork and social jetlag and is associated with CVD risks.^6^ Therefore, factors other than shiftwork and social jetlag may contribute to irregular sleep, but these are unclear.

The duration of the daily work interval (WI) between the end of one day’s working hours and the start of the next day’s working hours includes activities and/or behaviors normally performed outside of work hours, such as sleep, leisure time, commute time, and other nonwork time. Consequently, a longer WI could result in a longer sleep opportunity. In fact, previous research revealed that employees with a shorter daily rest period had significantly shorter sleep duration^18^ and extending the WI increases sleep quantity.^19^ Sleep duration is associated with several health problems.^1^^,^^2^^,^^4^ Therefore, ensuring a certain WI duration is important for worker health. The European Union (EU) working time directive stipulates that EU workers are entitled to take “a minimum daily rest period of 11 consecutive hours every 24 hr.”^20^ In Japan, a “work interval system” requires employers to ensure workers receive a certain number of hours between the end of one workday and the start of the next.^21^ Although these directives and systems mention WI duration, they do not address the interval’s regularity. In other words, even if the WI duration over 11 hours results in a certain sleep opportunity, irregular WI timing may occur, resulting in irregular sleep. However, whether WI regularity and sleep regularity are associated remains unclear.

Thus, this study examined the relationship between WI regularity and sleep regularity among nonshiftwork daytime workers. This study also hypothesized that irregular WIs would correlate with irregular sleep. Since this study focused on WI and sleep on workdays, nonworkday data were omitted from the analysis. In addition, the targeted population of this study was daytime workers in the “information and communications” industrial classification. Workers in this industry are assumed to have irregular work schedules, as this industry had the second-longest overtime work hours among all industry classifications in Japan,^22^ which may be due to its working characteristics of tight deadlines, customer support, and sudden changes in specification.^23^

## 2. Methods

### 2.1. Participants

This study recruited participants from October 5 to October 10, 2018, via an outsourced third-party research company with a database of voluntarily registered participants. The company sent invitations to 2731 workers (18-59 years old) in the information and communications industry who had previously registered. A total of 1066 registered users accessed the survey website and completed the screening survey (response rate: 39%). Participants were excluded based on the following criteria: (1) those who were not currently employed; (2) those who were shift or night workers; (3) those with nonpermanent employment (eg, part-time or temporary workers); and (4) those whose current industry type did not fall under “information and communications.” The 283 workers who met the aforementioned criteria were included in the observational study. All participants provided web-based informed consent. The study protocol was approved by the Research Ethics Committee of the National Institute of Occupational Safety and Health, Japan (H2916).

### 2.2. Procedures

The 14-day observational study was conducted from October 15 to October 28, 2018. During the observation period, participants were required to complete the survey daily before bedtime. Every evening, participants were emailed a survey. After the observation period, participants completed a post-survey regarding demographic data and their characteristics (eg, sleep quality) until November 2, 2018.

### 2.3. Measurements

Regarding WI variables, the start and end of the day’s working hours were requested. WI duration was determined as the time between the end of one day’s working hours and the start of the next day’s working hours. In addition, the midpoint of the WI (ie, WI timing) was calculated using the following formula: end of working hours + WI duration/2. Meanwhile, WI regularity was measured using the SD of the 14-day observation period in WI duration and WI timing. Since this study focused on the regularity of workday WIs, nonworkday WI data (ie, WI durations longer than 24 hours) were excluded from the calculation of regularity.

Regarding sleep variables, the previous bedtime and morning wake-up time were asked. The interval between bedtime and rise time was computed based on time in bed (TIB). The SD of the 14 observation days in TIB was used to quantify the regularity of sleep duration. In addition, the SD of bedtime over the 14-day observation period was used to quantify the regularity of sleep timing based on previous studies that used bedtime or sleep onset.^6^^,^^9^ The sleep data before and/or after the nonworkday (ie, data during WIs of >24 hours) were excluded from the calculation of regularity because the focus of this study was on the regularity of sleep on workdays.

### 2.4. Post-survey

The collected information about the participants’ characteristics included sex, height, weight (body mass index, BMI), one-way commute time, marital status (single or married), smoking status (current smoker, ex-smoker, or nonsmoker), and alcohol status (0, 1-2, 3-5, or ≥6 d/wk). In addition, the Japanese version of the Pittsburgh Sleep Quality Index (PSQI-J)^24^ was administered to assess the sleep quality. The PSQI-J consists of 18 questions, and the total score ranges from 0 to 21, which indicates sleep quality. The higher the score, the more severe the sleep complaints. The threshold for primary insomnia has been set at 5.5.

### 2.5. Analyses

Some participants may have given incorrect responses due to confusion between AM and PM. Therefore, in this study, “AM” and “PM” were changed to “PM” and “AM,” respectively, when confusion arose between “AM” and “PM” and when the answers met the following criteria: (1) the time order of the previous bedtime, wake-up time, start of working hours, and end of working hours was incorrect, and (2) the TIB and/or the WI duration (working hours) was disproportionately large or small. In addition, incorrect responses with the same time of day for bedtime and wake-up time and the same time of day for the start and end of working hours were eliminated.

Spearman correlation analysis was used to establish the relationships between sleep regularity (TIB SD and bedtime timing SD) and continuous variables. In addition, the Mann-Whitney *U* test was used to determine sleep regularity differences between groups of categorical variables. The variables that were significantly correlated with sleep regularity and showed significant differences between category groups were used as covariates in the following multivariate logistic regression analysis.

Since the TIB SD, bedtime timing SD, and log-transformed values were not normally distributed according to the Shapiro-Wilk test, multivariate logistic regression analysis was performed to explore the relationship between WI regularity (duration and timing) and sleep regularity (duration and timing). The dependent variables were the ≥60 minutes of TIB SD and ≥60 minutes of bedtime timing SD. The independent variables were WI duration SD and WI timing SD. However, due to a high correlation between WI duration SD and WI timing SD (spearman correlation coefficient *rs* = 0.930, *P* < .001), this study examined their associations with sleep regularity independently in all logistic regression analyses. WI duration SD (<30 min [*n* = 47], 30-60 min [*n* = 45], and ≥60 min [*n* = 49]) and WI timing SD (<30 min [*n* = 90], 30-60 min [*n* = 38], and ≥60 min [*n* = 13]) were used to categorize the participants. The <30 min (the most regular) group was chosen as the reference. In addition, the WI duration SD and WI timing SD data were set as continuous variables to independent variables based on the strengthening the reporting of observational studies in epidemiology (STROBE) statements.

Moreover, sensitivity analyses were performed to evaluate the robustness of the results. The above logistic regression analysis was repeated, focusing on workers who had never experienced a WI duration of <11 hours during the observation period.

Furthermore, Spearman correlation analysis was used to explore the relationships between WI regularity (WI duration SD and WI timing SD) and WI regularity-related factors (WI duration mean, WI timing mean, start of working hours mean, start of working hours SD, end of working hours mean, end of working hours SD, working hours mean, and working hours SD).

SPSS version 23.0 for Microsoft Windows was used to conduct all statistical analyses (SPSS Software Inc.).

## 3. Results

A total of 145 employees completed the 14-day observation survey and post-survey (response rate: 51%). Three participants who lacked bedtime and/or wake-up time data for more than 10 days and 1 participant who performed shiftwork (night work) for a week were excluded from further analyses. The final sample size included 141 employees who responded for all days for the 14-day observation period.

Table 1 presents the demographic data. The median TIB SD, median bedtime SD, median WI duration SD, and median WI timing SD were 0.5, 0.4, 0.7, and 0.4 hours, respectively.

**Table 1:** Demographic data (*n* = 141).

**Variables**	**Results**
Sex, female, *n* (%)	117 (83)
Age, median (IQR), y	47.0 (40.0-52.0)
Body mass index, median (IQR)	22.8 (20.6-24.6)
One-way commute time, median (IQR), h	1.0 (0.7-1.2)
Marital status, married, *n* (%)	53 (38)
Smoking status, smoker, *n* (%)	31 (22)
Alcohol status, ≥1/wk, *n* (%)	89 (63)
Time in bed, mean, mean (SD), h	6.3 (1.0)
Time in bed, SD, median (IQR), h	0.5 (0.3-0.7)
Bedtime, mean, mean (SD)	24.1 (1.2)
Bedtime, SD, median (IQR)	0.4 (0.3-0.8)
Wake-up time, mean, mean (SD)	6.4 (0.9)
Wake-up time, SD, median (IQR)	0.2 (0.0-0.4)
Work interval duration, mean, median (IQR), h	14.5 (14.0-15.0)
Work interval duration, SD, median (IQR), h	0.7 (0.4-1.3)
Work interval timing, mean, mean (SD)	25.8 (0.7)
Work interval timing, SD, median (IQR)	0.4 (0.2–0.7)
Start of working hours, mean, median (IQR)	9.0 (8.8-9.2)
Start of working hours, SD, median (IQR)	0.1 (0.0-0.4)
End of working hours, mean, median (IQR)	18.4 (18.0-19.1)
End of working hours, SD, median (IQR)	0.8 (0.4-1.2)
Working hours, mean, median (IQR), h	9.4 (9.1–10.0)
Working hours, SD, median (IQR), h	0.8 (0.4-1.4)
Pittsburgh Sleep Quality Index score, median (IQR)	5.0 (3.0-6.0)
Pittsburgh Sleep Quality Index cut off, >5, *n* (%)	50 (36)

Abbreviation: IQR, interquartile range.

Data are presented as mean (SD) for normally distributed variables or median (IQR) for nonnormally distributed variables, which is confirmed by the Kolmogorov-Smirnov test.

Regarding univariate analyses, the Mann-Whitney *U* test for TIB SD and bedtime timing SD showed no significant differences (*P* > .05) by sex (female or male), marital status (married or single), smoking status (smoker or non-/ex-smoker), alcohol status (nonconsumption or ≥1/wk), or PSQI cut-off score (≥6 or <6). In addition, the Spearman correlation analyses (Table 2) revealed that TIB SD was significantly correlated with WI duration SD (*rs* = 0.364, *P* < .001) and WI timing mean (*rs* = 0.211, *P* = .012) and SD (*rs* = 0.370, *P* = .001). Moreover, bedtime timing SD was significantly correlated with WI duration SD (*rs* = 0.366, *P* < .001) and WI timing mean (*rs* = 0.281, *P* = .001) and SD (*rs* = 0.372, *P* = .001). However, age, BMI, commute time, WI duration mean, and PSQI score were not correlated with TIB SD and bedtime SD (*P* > .05). As a result, the mean of WI timing was accounted for as a confounding factor.

**Table 2:** Results of the Spearman correlation analyses between sleep regularity (TIB SD and bedtime timing SD) and continuous variables.

	1	2	3	4	5	6	7	8	9	10
1. TIB SD	—									
2. Bedtime SD	0.832*	—								
3. Work interval duration SD	0.364*	0.366*	—							
4. Work interval timing SD	0.370*	0.372*	0.930*	—						
5. Work interval duration mean	−0.084	−0.133	−0.230*	−0.222*	—					
6. Work interval timing mean	0.211*	0.281*	0.183*	0.203*	−0.531*	—				
7. Age	−0.097	−0.094	−0.002	−0.079	0.064	−0.228*	—			
8. Body mass index	−0.035	0.070	0.164	0.148	−0.048	−0.020	0.292*	—		
9. Commute time	−0.073	−0.041	−0.058	−0.082	0.018	−0.014	0.172*	0.092	—	
10. Pittsburgh Sleep Quality Index score	0.040	0.045	0.203*	0.190*	−0.168*	0.047	−0.026	0.182*	0.120	—

Abbreviation: TIB, time in bed.

^*^ *P* < .05.

The numbers of workers with ≥60 minutes of TIB SD and bedtime timing SD were 24 and 27, respectively (Table 3). Regarding the WI duration SD, when the WI duration SD was set as a continuous variable, logistic regression analysis revealed that the crude odds ratio (OR) (95% CI) associated with a 1-hour increase in the WI duration SD was 2.509 (1.406-4.477) for ≥60 minutes of TIB SD and 2.432 (1.388-4.262) for ≥60 minutes of bedtime timing SD. When WI duration SD was set as a categorical variable, logistic regression analysis for ≥60 min of TIB SD revealed that, relative to the reference, the crude OR (95% CI) was 1.344 (0.337-5.360) for 30-60 minutes and 4.743 (1.441-15.607) for ≥60 minutes. In addition, logistic regression analysis for ≥60 minutes of bedtime timing SD revealed that the crude OR (95% CI) was 1.980 (0.538-7.293) for 30-60 minutes and 5.212 (1.592-17.061) for ≥60 minutes compared with the reference. These significant associations remained after adjusting for the confounding factor.

**Table 3:** Prevalence and odds ratios (95% CIs) for regularity of work interval and sleep (*n* = 141).

	**≥60 min of time in bed SD**			**≥60 min of bedtime timing SD**		
		**Odds ratios (95% CIs)**			**Odds ratios (95% CIs)**	
	**Prevalence**	**Crude**	**Adjusted** ^**a**^	**Prevalence**	**Crude**	**Adjusted** ^**a**^
*Work interval duration SD*						
Continuous, h	24/141	**2.509 (1.406-4.477)**	**2.488 (1.368-4.524)**	27/141	**2.432 (1.388-4.262)**	**2.447 (1.348-4.443)**
Categorical						
<30 min	4/47	ref	ref	4/47	ref	ref
30-60 min	5/45	1.344 (0.337-5.360)	1.300 (0.321-5.258)	7/45	1.980 (0.538-7.293)	1.969 (0.515-7.528)
≥60 min	15/49	**4.743 (1.441-15.607)**	**4.303 (1.285-14.414)**	16/49	**5.212 (1.592-17.061)**	**4.770 (1.398-16.268)**
*Work interval timing SD*						
Continuous, h	24/141	**8.911 (2.667-29.776)**	**8.096 (2.366-27.711)**	27/141	**9.301 (2.850-30.348)**	**8.391 (2.443-28.816)**
Categorical						
<30 min	8/90	ref	ref	9/90	ref	ref
30-60 min	10/38	**3.661 (1.315-10.192)**	**3.332 (1.178-9.424)**	12/38	**4.154 (1.574-10.965)**	**3.737 (1.371-10.187)**
≥60 min	6/13	**8.786 (2.371-32.561)**	**7.829 (2.033-30.139)**	6/13	**7.714 (2.124-28.015)**	**6.721 (1.696-26.637)**

Odds ratios are based on logistic regression analysis. Significant odds ratios (*P* < .05) and 95% CIs are presented in bold.

a Adjusted for the mean of work interval timing.

Regarding the WI timing SD, logistic regression analysis revealed that the crude OR (95% CI) associated with a 1-hour increase in the WI timing SD was 8.911 (2.667-29.776) for ≥60 minutes of TIB SD and 9.301 (2.850-30.348) for ≥60 minutes of bedtime timing SD. When the WI timing SD was set as a categorical variable, logistic regression analysis for ≥60 minutes of TIB SD revealed that the crude OR (95% CI) was 3.661 (1.315-10.192) for 30-60 minutes and 8.786 (2.371-32.561) for ≥60 minutes compared with the reference. In addition, logistic regression analysis for ≥60 minutes bedtime timing SD revealed a crude OR (95% CI) of 4.154 (1.574-10.965) for 30-60 minutes and 7.714 (2.124-28.015) for ≥60 minutes compared with the reference. These significant associations remained after adjusting for the confounding factor.

Table 4 shows the results of sensitivity analysis, focusing only on workers who had never experienced a WI duration of <11 hours during the observation period (*n* = 133). Similar relationships existed between WI regularity and sleep regularity.

**Table 4:** Sensitivity analysis: prevalence and odds ratios (95% CIs) for the regularity of the work interval and sleep (*n* = 133).^a^

	**≥60 min of time in bed SD**			**≥60 min of bedtime timing SD**		
		**Odds ratios (95% CIs)**			**Odds ratios (95% CIs)**	
	**Prevalence**	**Crude**	**Adjusted** ^**b**^	**Prevalence**	**Crude**	**Adjusted** ^**b**^
*Work interval duration SD*						
Continuous, h	20/133	**2.178 (1.127-4.210)**	**2.427 (1.200-4.905)**	23/133	**2.174 (1.154-4.097)**	**2.667 (1.307-5.444)**
Categorical						
<30 min	4/47	ref	ref	4/47	ref	ref
30-60 min	5/45	1.344 (0.337-5.360)	1.301 (0.319-5.301)	7/45	1.980 (0.538-7.293)	2.033 (0.517-7.995)
≥60 min	11/41	**3.942 (1.146-13.563)**	**4.096 (1.156-14.514)**	12/41	**4.448 (1.306-15.153)**	**5.230 (1.411-19.387)**
*Work interval timing SD*						
Continuous, h	20/133	**6.064 (1.613-22.795)**	**6.162 (1.558-24.368)**	23/133	**6.867 (1.899-24.834)**	**7.793 (1.900-31.974)**
Categorical						
<30 min	8/90	ref	ref	9/90	ref	ref
30-60 min	8/33	**3.280 (1.117-9.634)**	**3.330 (1.114-9.952)**	10/33	**3.913 (1.421-10.772)**	**4.340 (1.488-12.655)**
≥60 min	4/10	**6.833 (1.589-29.384)**	**6.575 (1.436-30.103)**	4/10	**6.000 (1.421-25.335)**	**5.936 (1.178-29.910)**

Odds ratios are based on logistic regression analysis. Significant odds ratios (*P* < .05) and 95% CIs are presented in bold.

a Workers who had work intervals <11 h were excluded from the analysis.

b Adjusted for the mean of the work interval timing.

The results of the Spearman correlation analyses between WI regularity and the factors associated with it are shown in Table 5. The WI duration SD was significantly correlated with WI duration mean (*rs* = −0.230, *P* = .006), WI timing mean (*rs* = 0.183, *P* = .03), start of working hours SD (*rs* = 0.416, *P* < .001), end of working hours mean (*rs* = 0.211, *P* = .012), end of working hours SD (*rs* = 0.827, *P* < .001), working hours mean (*rs* = 0.249, *P* = .003), and working hours SD (*rs* = 0.882, *P* < .001). Moreover, the WI timing SD was significantly correlated with WI duration mean (*rs* = −0.222, *P* = .008), WI timing mean (*rs* = 0.203, *P* = .016), start of working hours SD (*rs* = 0.410, *P* < .001), end of working hours mean (*rs* = 0.248, *P* = .003), end of working hours SD (*rs* = 0.832, *P* < .001), working hours mean (*rs* = 0.258, *P* = .002), and working hours SD (*rs* = 0.843, *P* < .001).

**Table 5:** Results of the Spearman correlation analyses between work interval regularity and factors related to work interval regularity.

	1	2	3	4	5	6	7	8	9	10
1. Work interval duration, SD	—									
2. Work interval timing, SD	0.930^*^	—								
3. Work interval duration, mean	−0.230^*^	−0.222^*^	—							
4. Work interval timing, mean	0.183^*^	0.203^*^	−0.531^*^	—						
5. Start of working hours, mean	0.011	0.023	0.181^*^	0.637^*^	—					
6. Start of working hours, SD	0.416^*^	0.410^*^	−0.068	0.051	0.009	—				
7. End of working hours, mean	0.211^*^	0.248^*^	−0.735^*^	0.899^*^	0.361^*^	0.041	—			
8. End of working hours, SD	0.827^*^	0.832^*^	−0.214^*^	0.194^*^	0.030	0.295^*^	0.223^*^	—		
9. Working hours, mean	0.249^*^	0.258^*^	−0.940^*^	0.556^*^	−0.158	0.098	0.811^*^	0.227^*^	—	
10. Working hours, SD	0.882^*^	0.843^*^	−0.215^*^	0.182^*^	0.014	0.452^*^	0.192^*^	0.919^*^	0.226^*^	—

^^*^^ *P* < .05.

## 4. Discussion

This study examined the relationship between WI regularity (duration and timing) and sleep regularity (duration and timing) in daytime workers without shiftwork. Results indicated that irregular WI duration and timing were significantly associated with irregular sleep duration and timing, and these associations persisted when analyzing only the participants who had never experienced a WI duration of <11 hours during the observation period. These results suggest that day-to-day variation in WI duration and/or timing is associated with sleep regularity in the absence of shiftwork and the gap between workdays and nonworkdays, both of which have a substantial impact on sleep regularity.

This result supports our hypothesis that irregular WI duration and timing are associated with irregular sleep duration and timing. WI includes the sleep opportunity, and irregular WI duration may result in irregular sleep opportunities. According to a previous study, the average WI duration in the preceding month was correlated with the average TIB in the preceding month,^18^ and it is believed that if the WI duration varies from day to day, sleep duration may also vary, leading to irregular sleep. In addition, WI timing regularity was associated with sleep timing regularity, meaning that irregular WI timing is associated with irregular sleep timing. This result may be attributable to irregular WI timing that includes sleep opportunities that may also result in irregular sleep timing. Previous studies also reported that shiftwork, especially with the night shift, also causes irregular sleep/wake schedules (timing)^15^ and poses a risk of several health problems such as fatigue, sleep problems, and CVD.^16^^,^^17^^,^^25^ This study newly found that irregular WI timing without shiftwork may also be associated with an increased risk of irregular sleep timing. Huang et al^6^ found that irregular sleep duration and timing were associated with cardiovascular events, and the relationship remained when shift workers were excluded. In addition, previous studies have reported that regular sleep is associated with many health issues.^6–10^ Therefore, even daytime employees who do not work shifts should take regular WIs.

WI duration and timing were also correlated with sleep timing and duration, respectively. Sleep duration regularity was highly correlated with regularity of sleep timing, and this result was consistent with a previous study,^26^ suggesting that when workers shift their sleep duration, they also shift their sleep timing. In addition, there was a strong correlation between regularity of WI duration and regularity of WI timing. This association may explain the significant correlation between regularity of WI duration and regularity of sleep timing, and between regularity of WI timing and regularity of sleep duration.

Furthermore, sensitivity analysis showed that the significant relationship between WI regularity and sleep regularity remained when participants with WI durations of <11 hours were excluded. These results suggest that even if the workers could ensure a WI of ≥11 hours as per the EU working time directive (minimum daily rest period of 11 consecutive hours every 24 hours)^20^ or the WI system in Japan,^21^ their sleep regularity may become irregular. In addition, irregular sleep causes a number of health problems.^6–10^ As a countermeasure, workers should avoid irregular duration and timing of WIs as much as possible. In addition, workers with irregular WIs should alter their sleep duration and timing as little as possible.

This study focused on WI, which includes sleep opportunities, and examined the relationship between sleep regularity and WI regularity. Working hours and WI were calculated from the start and end of working hours, and the relationship between WI and working hours is like 2 sides of the same coin. As a result, the working hours SD was highly correlated with WI regularity and may be related to sleep regularity. Furthermore, WI regularity was correlated with the start and end of working hours SD, in particular, with the end of working hours SD. Many workers who do not use the flextime or discretionary labor systems may have a fixed start time for work. As a result, the irregular WI and irregular sleep reported in this study could be attributed primarily to the irregular end of working hours. Meanwhile, if workers who use the WI system finish their shifts late and the WI falls below 11 hours, their start time for the next day will be delayed as well. This indicates that, compared with workers with a fixed start of working hours, the WI system can result in a certain duration of the WI but delay the phase of WI due to a delayed start in working hours, causing irregular WI. Therefore, workers under the WI system should try to maintain as much sleep duration and timing as possible.

This study has a number of limitations. First, a web survey was used to collect data for this study, which may have introduced sampling bias. Second, self-reports were used to measure the start and end of working hours (WI duration and timing), bedtime, and wake-up time (TIB and sleep timing). Additional research employing objective measures such as attendance record (objective work schedules) and actigraphy (objective sleep variables) is required. Third, this study was conducted in 2018, that is, before the COVID-19 pandemic. Following COVID-19, the work-from-home setting has been rapidly and widely implemented in recent years, particularly in Japan’s “information and communications” industry.^27^ Working from home can reduce commuting time, affecting sleep duration and regularity. However, no information about working from home was requested in this study. More research is needed to consider the effects of the work-from-home system on sleep duration and regularity. Fourth, this study did not inquire about a history of CVD events linked to irregular sleep.^6^ As irregular WI was linked to irregular sleep, irregular WI may be indirectly linked to CVD events through irregular sleep. More research is needed to clarify this relationship.

### 4.1. Conclusion

Regularity of WI duration and timing was related to regularity of sleep. This result was also found among workers who had not experienced WI durations of <11 hours, indicating that although the EU working time directive or the WI system in Japan can guarantee a specific WI duration, irregular sleep may not be prevented. Irregular sleep leads to several health problems. Therefore, to protect worker health, workers should maintain regular WIs, and if they experience irregular WIs, they may need to maintain as regular a sleep schedule as possible.

## Data Availability

The data underlying this article will be shared on reasonable request to the corresponding author.
